# Difficulties in assessing the medical fitness of workers with schizophrenia

**DOI:** 10.1192/j.eurpsy.2026.12133

**Published:** 2026-07-31

**Authors:** W. Ayed, S. Chemingui, Y. Sellaouti, M. Mersni, G. Bahri, M. Bani, H. Nefzi, N. Ladhari

**Affiliations:** 1 Occupational Medicine Department, Charles Nicolle Hospital, Tunis; 2 Psychiatric G, Razi hospital, Mannouba, Tunisia

## Abstract

**Introduction:**

Schizophrenia is a chronic psychiatric disorder that often emerges during early adulthood and can significantly affect social, professional, and functional outcome

**Objectives:**

- To assess the medical and occupational characteristics of workers with schizophrenia .
- To evaluate the impact of these psychiatric disorders on the medical decision of fitness for work.

**Methods:**

Descriptive and retrospective study, over five years (January 1, 2020 to June 30, 2025) including all medical records of workers with schizophrenia, referred to the occupational department of the Charles-Nicolle Hospital in Tunis for a medical fitness for work.

**Results:**

The study included 22 patients, mostly male (sex ratio (M/F) = 2,6), with a mean age of 44 ± 7 years. The most represented sector of activity were the administrative department (55%) and health care (32%). The participants were mainly blue-collar workers (37%), followed by nurses (20%). The mean job seniority was 14 ± 7 years. The symptoms reported by patients were hallucinations (53%), bizarre behaviour (13%) and sleep disorders (13%). They were all undergoing antipsychotic medical treatment. Concerning the fitness for work, three patients were fit for work with exclusion from positions of responsibility and 14 were temporarily unfit. Job mutation was recommended for one patient. Early retirement due to invalidity was proposed for two pat

**Conclusions:**

Schizophrenia has a considerable impact on workers’ fitness for work, often requiring temporary unfitness, job adjustments, or early retirement. Occupational health evaluation plays a key role in balancing patient protection, workplace safety, and job retention through tailored recommendations.

**Disclosure of Interest:**

None Declared

